# Editorial: Woody Plants and Forest Ecosystems in a Complex World—Ecological Interactions and Physiological Functioning Above and Below Ground

**DOI:** 10.3389/fpls.2020.00173

**Published:** 2020-02-28

**Authors:** Boris Rewald, Christian Ammer, Henrik Hartmann, Andrey V. Malyshev, Ina C. Meier

**Affiliations:** ^1^Forest Ecology, Department of Forest and Soil Sciences, University of Natural Resources and Life Sciences Vienna (BOKU), Vienna, Austria; ^2^Silviculture and Forest Ecology of the Temperate Zones, University of Göttingen, Göttingen, Germany; ^3^Department of Biogeochemical Processes, Max Planck Institute for Biogeochemistry, Jena, Germany; ^4^Experimental Plant Ecology, Institute of Botany and Landscape Ecology, University of Greifswald, Greifswald, Germany; ^5^Plant Ecology, Albrecht-von-Haller Institute of Plant Sciences, University of Goettingen, Göttingen, Germany

**Keywords:** competition, disturbance, functional traits, forest ecosystem research, funding priorities, resource availability, stressors, trees

Forests are fundamental components of life on Earth. They are essential to global biogeochemical cycles and economies alike, shape the landscape, provide habitat for a large number of animal and plant species, are a renewable resource of wood, and provide a carbon sink that can reduce anthropogenic CO_2_ pollution and mitigate climate change (FAO, 2018b). Yet despite the importance of intact forest ecosystems for future generations of humankind (Costanza et al., 1997), notoriously more research funds are directed towards agricultural systems, or wood processing at most (Lovrić et al., 2020), than towards the preservation and sustainable management of forest ecosystems under global change. This is alarming, as in times of rapidly changing environmental conditions, resource management of long-lived woody species and their ecosystems is facing new challenges (Macdicken et al., 2016; Ammer, 2019). The Intergovernmental Science-Policy Platform on Biodiversity and Ecosystem Services recently underscored that critical knowledge gaps remain for (i) ecosystem processes that underpin the contribution of nature to people and ecosystem health as well as for (ii) the consequences of changing interactions among organisms and taxa under climate change (IPBES, 2019). These knowledge gaps strongly apply to forest ecosystems. Here we call for a reconsideration of economic funding decisions that should jointly take into account forest-based bioeconomy (Kraxner et al., 2017), global biogeochemical cycles (Curtis and Gough, 2018), as well as various other regulating, ecological, cultural, and supporting services provided by forest ecosystems (Felipe-Lucia et al., 2018). This implies the explicit inclusion of (pristine and managed) forest ecosystems, incl. their soils, into the EUs’ Horizons Europe research program (European Commission, 2019) and other national and supranational funding schemes worldwide.

We support our call by the large number of open questions related to forest ecology [see e.g. (Ammer et al., 2018) for Central Europe] and recent research on woody species and forest ecosystems. The 18 articles in this Research Topic (RT) not only uncover ecological interactions and physiological functioning above- and belowground, but also illustrate specific open questions and research gaps that need to be covered—allowing for better predictions of forest responses to global change and other human activity-based ecosystem modifications. The better predictions are urgently needed when it comes to adapt forest management and ensure the provision of ecosystem services under global change (Joyce et al., 2009; Keenan, 2015).

Functional traits are increasingly recognized as a useful tool to understand structure–function relationships in plants (Díaz et al., 2016; O’brien et al., 2017). In this RT, aboveground plant functional traits are shown to be useful in estimating and predicting water use efficiency, carbon stocks and competitive ability due to their inherent conservative nature. Specifically, Wang et al. report on the existence of a cross-species trade-off between the size of individual leaves and the number of leaves per yearly twig unit in a temperate forest and provide evidence for the influence of small leaf sizes on effective temperature regulation in dry environments with strong radiation. As the relationships between leaf and twig biomass as well as between leaf biomass and twig and stem dimensions are important due to their relation to plants’ C metabolism and transport modes, Sun et al. focus on isometric scaling across different forest communities in the humid warm subtropics. The article by Bu et al. illustrates that plant functional traits such as specific leaf area and wood density, as affected by abiotic site conditions in a tropical montane rainforest, are key in estimating aboveground carbon stocks. Their study suggests that community-weighted means of plant functional traits are key mediators in regulating effects of abiotic site conditions on ecosystem functions. Complementary, the analysis of *Pinus sylvestris* fine-root traits by Brunner et al. reveals that a long-term increase in water availability significantly increases root biomass, length, and production, but does not alter morphological and architectural traits. Strikingly, their experiment illustrates the extended time spans necessary to reveal acclimation responses in trees—the differences in fine-root biomass between irrigated and dry plots became significant only nine years after the experiment began. This strongly suggests that more long-term experiments are needed in forest research addressing the impact of changing environments.

In dense communities, plants compete with each other for limited resources. Assessing the neighborhood of tree saplings with terrestrial laser scanners, Annighöfer et al. show that individuals of the same species perform differently under constant light conditions but differing neighborhoods. Their study confirms that the description of the neighborhood, in addition to light measurements, increases the predictive power of tree regeneration trait and performance models. However, while the amount of light is considered to be among the most important resources for saplings growing in the understory of (temperate) forest ecosystems, Bueno et al. illustrate that competition for nitrogen (N) can also modify the outcome of competitive interactions between invasive and native tree seedlings. Other articles in this RT address resource gathering and allocation patterns of woody species and their subsequent effects on growth. Assessing phosphorous (P) nutrition under continuing high N deposition and increasing drought stress, respectively, Meller et al. highlight the ability of *Fagus sylvatica* to alter resource allocation in response to soil P availability, while Dirks et al. foresee growth reductions in *Quercus calliprinos* trees due to reduced P uptake efficiency with increased drought occurrence. As plant resource uptake (capacity) is often tightly related to ectomycorrhizal fungi (EMF) in temperate and boreal forest ecosystems, identifying the biotic and abiotic factors that shape EMF communities is important for understanding terrestrial ecosystem processes and predicting the impacts of global change on plant communities. Accordingly, Defrenne et al. shed light on the ectomycorrhizal fungal diversity in *Pseudotsuga menziesii* forests across regional gradients in Canada. They find that temperature, precipitation, and the soil C:N ratio affect EMF community dissimilarities and exploration type abundances. As no evidence for a functional connection between root diameter and EMF exploration types within Douglas-fir populations was found, future studies are encouraged to simultaneously examine both fine root and fungal traits—representing the diversity of belowground resource gathering strategies.

Focusing on plant–water relations in a subtropical ecosystem, Fang et al. suggest that bamboos exchange water *via* rhizomes and that nighttime fluxes are highly important for the support of freshly sprouted culms. The study provides further insight into the benefits of physiological integration in woody monocots; water exchange may facilitate the very fast growth of bamboo shoots and the colonization of the surroundings. Gong et al. (2019) provide an example on the significance of foliar water uptake for the water balance under arid conditions—even in a deep-rooted tree species. Geißler et al. measured soil moisture dynamics at different depths with natural stable isotopes to quantify the partitioning of water between vegetation components. Consequently, potential effects of shrub encroachment on groundwater recharge rates in a climate-change affected semi-arid savanna ecosystem were determined. Finally, Martín-Sanz et al. investigated the effect of water availability on the resilience to forest fires in 19 *Pinus halepensis* provenances *via* absolute bark thickness (i.e. determining the degree of heat insulation). They show that drought-stressed trees have a higher risk to die from fires before achieving sexual maturity and building a sufficiently large aerial seed bank for post-fire regeneration. As forest fires are suggested to increase in frequency and extent, especially in the Mediterranean areas, the results highlight the unfortunate interactions between different stress and disturbance agents.

Together with forest fires, pest infestations have caused large-scale disturbances of (temperate) forests in recent years. Erfmeier et al. illustrate that younger ash trees, particularly those growing in alder-ash forests, are most susceptible to Ash dieback—thus showing that forest community composition and age play key roles in the extent of the infection with *Hymenoscyphus fraxineus*. In a broader perspective, Rammer and Seidl argue for an increased utilization of the predictive power of machine learning techniques such as deep neural networks for a wide range of ecological problems—using bark beetle outbreaks in conifer-dominated forests as an example.

Shifting the focus towards disturbances of smaller scale, wind damage is an important factor among temperate forests (Sommerfeld et al., 2018). Dumroese et al. thus address the plasticity of *Pinus ponderosa* coarse root (systems) as a function of prevailing wind and sloping ash-cap soils, improving our functional understanding of tree anchorage. An enhanced knowledge on anchorage properties of trees will enable us to better predict the response of trees to more severe, more frequent climate change-facilitated storms, as well as to inform silvicultural practices toward improving the resilience of existing forest stands towards multiple stressors (Bolte et al., 2019). While climate change affects all seasons, the ecological effects of warming are often more pronounced in winter than summer (Kreyling et al., 2019)—i.a. leading to insufficient/pre-mature (de-)hardening with an increased risk of frost damage (Inouye, 2008). Addressing frost survival mechanism of vegetative buds in temperate trees, Neuner et al. show that the mechanism of frost resistance in temperate trees helps explain which species can withstand lower freezing temperatures and how they are distributed. The ability to withstand intracellular freezing is present in species with the best ability to withstand freezing damage at the lowest temperatures.

Green infrastructure becomes more and more important in an increasingly urbanized world (FAO, 2018a). Management of urban forests is a challenging task not only because of highly heterogeneous, often harsh growing conditions (Fitzky et al., 2019) but also because of various, often conflicting, demands and goals. In their contribution, Vitali et al. illustrate that damaging root systems of urban trees invokes long-term effects on growth and that slower growing tree species can better compensate growth reductions due to the administered damage. The paper highlights the potential of increased species functional understanding to manage (future) vegetation composition in urban environments better.

Taken together, the 18 contributions to this RT cover many aspects of timely research on woody plants and forest ecosystems. They add to the increasing evidence that one-size-fits-all tree species-to-forest type ecosystem services relationships are overly simplistic, arguing for a more nuanced empirical understanding of forest functioning under different environmental and management regimes. However, the current dominance of funding for bio-energy and bio-refinery topics over more forest ecosystem-focused studies [e.g. in the EU; (Lovrić et al., 2020)] follows the perspective of science and technology studies (Birch et al., 2010)—by which bio-economy research is focused on the innovation potential of biotechnology, and less on the sectors that manage natural resources. In contrast, we believe that an increased investment into cross-disciplinary ecological research is needed to strengthen our functional understanding of woody plants in complex ecosystems. Especially assessments of resilience and adaptive capacity of forest ecosystems under a changing climate remain important priorities of research to support evidence-based decisions by forest managers and policy makers worldwide on sustainable management practices.

## Author Contributions

BR, IM and AM provided the first draft of the MS. All co-authors jointly revised the manuscript and approved its publication.

## Conflict of Interest

The authors declare that the research was conducted in the absence of any commercial or financial relationships that could be construed as a potential conflict of interest.
